# Medical education in Malaysia: quality versus quantity

**DOI:** 10.1007/s40037-016-0319-8

**Published:** 2017-01-03

**Authors:** Rebecca S. Y. Wong, Samiah Yasmin Abdul Kadir

**Affiliations:** Faculty of Medicine, SEGi University, Kota Damansara, Selangor Malaysia

Medical education in Malaysia has a long history dating back to the establishment of the Faculty of Medicine at the University of Malaya in 1963 [1]. Currently, there are 32 medical schools in the country (11 public and 21 private). At the end of 2014, there were 18,789 students in all years in Malaysian medical schools and another estimated 15,000 Malaysians studying medicine abroad [2]. This implies that 30,000 doctors will join the Malaysian healthcare system within the next five years and by 2018 the number of doctors will double [2]. The World Health Organisation’s (WHO) recommendation of 1 doctor to 400 persons for a developed nation has frequently been used to justify this mass production of doctors.

While having too many doctors can be problematic, having too many poor-quality doctors can certainly worsen the problem. Since 2009, 20% of the medical students who enter foreign universities to study medicine lack the minimum entry qualifications [3] and news on the poor quality of medical graduates is not uncommon in the mainstream media of Malaysia. Another issue concerning the proliferation of medical schools, particularly in the past decade, is the quality of the schools themselves. In recent years there have been reports of smaller or less stable private medical schools facing financial problems, with some of them closing down or having their international partners withdraw their collaboration.

To combat the issue of quantity, the government announced a five-year moratorium (from 2011 to 2016) on medical programmes in 2010 [4], whereas in 2014, the number of students being accepted into public medical schools has decreased. Besides, some consolidation has already taken place in the industry with the smaller players closing down, downsizing or the merging of the smaller campuses into bigger ones [5].

On the other hand, the extension of the duration of internship from one to two years started in 2008. While this is supposed to address the issue of quality, it has contributed to yet another problem. With the rising number of medical graduates and the extension of internship, the public hospitals are now flooded with house officers. More recently, in 2015, the government announced that the Ministry of Health will no longer arrange internship placements and that medical graduates are to apply to the government hospitals that offer internship themselves [6]. This may result in a longer wait for internship as there are limited vacancies and an increasing number of medical graduates.

With a handful of burning issues facing the healthcare system and medical education in Malaysia, we propose that the following actions are taken to improve the current situation:

- A strategic long-term plan: A strategic ten-year or longer-term plan for medical education in Malaysia is crucial. There must be a more organized way of imposing and monitoring changes in the healthcare and medical education systems.
- Adhering to global standards: Malaysian medical schools should refer to the standards set by the World Federation for Medical Education (WFME). The WFME started a programme on the international standards in medical education in 1997, which has been supported by the WHO/WFME Strategic Partnership to improve medical education since 2004 [7]. These standards, which can be modified to suit the Malaysian context, are very useful as they cover many important areas of medical education [8], from the national regulators to the curriculum developers, and those responsible for the assessment and accreditation of the programmes.
- Accreditation of medical schools using the WFME programme: Although the WFME and WHO are not accreditation bodies, they provide a programme for assisting medical schools, national agencies and authorities in establishing accreditation of basic medical education [9].
- Raising the bar: Not only should the entry requirements be raised (especially for the private medical schools), the exit requirements should also be raised. This is to ensure high-quality students get selected into the course and only high-quality students are allowed to graduate from medical schools. The WFME recommends that the number and nature of examinations should encourage the acquisition of the knowledge base and integrated learning. Both the reliability and validity of the assessment methods are important, and medical schools should ensure that these assessments are open to scrutiny by external expertise [8].
- Lengthening the medical course: For schools that run a five-year undergraduate medical programme, extending the course to six years may allow students to be more adequately trained before they start practising medicine. Again, the WFME standards may be referred to with regards to the programme structure, composition and duration. Not only should students be adequately trained, the WFME places emphasis on a horizontal integration of basic medical sciences and a vertical integration between basic medical sciences and clinical sciences [8].
- Merging or consolidation of the smaller medical schools: It may be necessary to further merge or consolidate the smaller medical schools to avoid a repeat of the current situation with some medical schools in financial crisis.
- Career counselling: This should be given to students towards the end of their secondary school education. Both students and parents need to be informed of other equally good options besides medicine. For those who have graduated from medical school, they should be exposed to other career options such as medical researcher, medical lecturer or medical advisor in pharmaceutical companies.
- Allowing internship and the two-year compulsory government service to be carried out in credible private hospitals: This can divert house officers and medical officers to the private hospitals. However, the for-profit nature of private hospitals and the patients may not welcome this concept.
- Redistribution of house officers and medical officers from urban to rural areas: With an oversupply of doctors in some parts of Malaysia and a longer wait in obtaining an internship placement, the government should consider making part of the internship (e. g. six months out of the two years) compulsory in the rural areas.
- Increase in the intake of medical officers into specialization with shortage of manpower: Instead of competing for places in popular specialties, the government can divert medical officers to areas where there is limited manpower.

It is time for medical education in Malaysia to change strategy. This requires the Ministry of Education, the Ministry of Health, the Malaysian Medical Council and the Malaysian Qualifications Agency to work closely on a feasible long-term strategic plan for medical education in Malaysia before things become out of control.
